# Pulmonary artery-targeted low-dose metformin-loaded nanocapsules safely improve pulmonary arterial hypertension in rats

**DOI:** 10.3389/fphar.2025.1577570

**Published:** 2025-04-30

**Authors:** Ayako Chida-Nagai, Naoki Masaki, Hiroki Sato, Tatsuya Kato, Emi Takakuwa, Yoshihiro Matsuno, Atsushi Manabe, Atsuhito Takeda

**Affiliations:** ^1^ Department of Pediatrics, Hokkaido University Hospital, Sapporo, Japan; ^2^ Department of Pediatric Cardiology and Adult Congenital Cardiology, Tokyo Women’s Medical University, Tokyo, Japan; ^3^ Division of Cardiovascular Surgery, Tohoku University Graduate School of Medicine, Sendai, Japan; ^4^ Department of Cardiology and Clinical Examination, Oita University, Yufu, Japan; ^5^ Advanced Trauma, Emergency and Critical Care Center, Oita University Hospital, Yufu, Japan; ^6^ Department of Thoracic Surgery, Hokkaido University Hospital, Sapporo, Japan; ^7^ Department of Surgical Pathology, Hokkaido University Hospital, Sapporo, Japan

**Keywords:** pulmonary arterial hypertension, metformin, liposome, nanocapsule, drug delivery system

## Abstract

**Introduction:**

Pulmonary arterial hypertension (PAH) remains a challenge to tackle despite various available medications. Metformin, although promising, has major adverse effects; the use of an appropriate drug delivery method may improve its efficacy and safety. The aim of this study was to develop a novel treatment for PAH using metformin. We developed a novel approach of using low-dose metformin encapsulated in pulmonary artery-targeted nanocapsules to alleviate PAH while avoiding adverse effects.

**Methods:**

Metformin-loaded lung-targeted nanocapsules (MET nanocapsules) were created using a specific lipid composition, including cationic lipids. Their uptake and effects on cell viability were assessed in human pulmonary arterial smooth muscle cells (hPASMCs) from healthy individuals and patients with PAH. Their therapeutic effects were assessed in a PAH rat model. The safety of MET nanocapsules was confirmed using rat serum biochemical tests.

**Results:**

We successfully prepared MET nanocapsules and demonstrated their effectiveness in inhibiting PASMC proliferation. In PAH model rats, MET nanocapsule treatment led to improved hemodynamics, right ventricular hypertrophy, and pulmonary arterial medial thickening. The nanocapsules effectively accumulated in the lungs of PAH model rats.

**Conclusion:**

Intravenous administration of MET nanocapsules is a safe and innovative therapeutic approach for PAH. This method could improve PAH treatment outcomes while minimizing adverse effects, with potential applications in other types of pulmonary hypertension.

## 1 Introduction

Pulmonary arterial hypertension (PAH) is a pathological condition characterized by elevated vascular resistance in the small pulmonary arteries, leading to a resting mean pulmonary arterial pressure exceeding 20 mmHg (Humbert et al., 2022). Despite the availability of various pulmonary vasodilators, such as prostacyclin analogs, phosphodiesterase five inhibitors, and endothelin receptor antagonists, the prognosis of PAH remains unfavorable, with a 5-year survival rate of approximately 60% (Boucly et al., 2021; Farber et al., 2015).

In PAH, the levels of inflammatory cytokines in the pulmonary arteries, including IL-6 and IL-1β, increase, contributing to pulmonary arterial remodeling and disease exacerbation (Rong et al., 2022; Selimovic et al., 2009). The AMPK enzyme reportedly inhibits PAH development (Omura et al., 2016; Shen et al., 2020).

Metformin, originally used for treating type 2 diabetes, has various biological effects, including inhibiting glucose release from the liver and increasing insulin sensitivity in peripheral tissues, particularly the muscles (Foretz et al., 2019). Owing to metformin’s versatile effects, its efficacy in treating other diseases has been explored. Metformin reduces the incidence of cancer in patients with type 2 diabetes, implying a potential anti-tumorigenic role (Morales and Morris, 2015). AMPK, an essential enzyme responsible for maintaining vital cellular functions, is activated by metformin (Lv and Guo, 2020). Metformin suppresses cancer development by activating AMPK in various organs, including the liver.

Based on these findings, several studies, including clinical trials (Brittain et al., 2020), have examined whether metformin can be used for treating PAH (Dean et al., 2016; Omura, et al., 2016; Zhai et al., 2018). However, the safety of metformin in patients with PAH, particularly in pediatric and older patients, as well as patients with liver or kidney dysfunction, remains unclear. In addition, metformin is associated with major adverse effects, including life-threatening lactic acidosis, hypoglycemia, gastrointestinal disturbances, and hepatic impairment (Zibar and Zibar, 2017).

We hypothesized that the use of a drug delivery system can maximize the effectiveness and safety of metformin in PAH treatment. Therefore, we developed a novel therapeutic approach, combining low-dose metformin with pulmonary artery-targeted nanocapsules, with the aim to alleviate PAH while avoiding the adverse effects associated with conventional metformin therapy.

## 2 Materials and methods

Metformin-loaded nanocapsules were prepared using the thin-film hydration method. Dioleoyl phosphatidylcholine (40 mol; COATSOME ME-8181; NOF Co., Tokyo, Japan), cholesterol (45 mol; cholesterol HP; NOF Co.), diocetyl phosphate (DCP; 5 mol; Sigma-Aldrich, St. Louis, MO, United States), and dioleoyl phosphatidylethanolamine (DOPE; 10 mol; COATSOME MC-8181, NOF Co.) were mixed to prepare a lipid film. A cationic lipid (dendron-bearing lipid D12; Katayama Chemical Co., Osaka, Japan) was added at 2 mol% of the total lipids to improve the delivery rate to the lungs (Cheng et al., 2020). We prepared the nanocapsules following established protocols (Bangham and Horne, 1964; Zhang, 2017). Lipids were weighed and transferred into a pear-shaped flask, to which a 1:1 (v/v) mixture of methanol:chloroform was added. This mixture was then stirred at 37°C for 1 h. Subsequently, the flask was attached to a rotary evaporator (N-1000; EYELA, Tokyo, Japan), and the solvents were evacuated in a nitrogen atmosphere at 37°C. Finally, the residue was placed in a desiccator and subjected to vacuum drying for 1 h using a PHIL P165D vacuum pump (SATO VAC Inc., Miyoshi, Japan).

**FIGURE 1:**
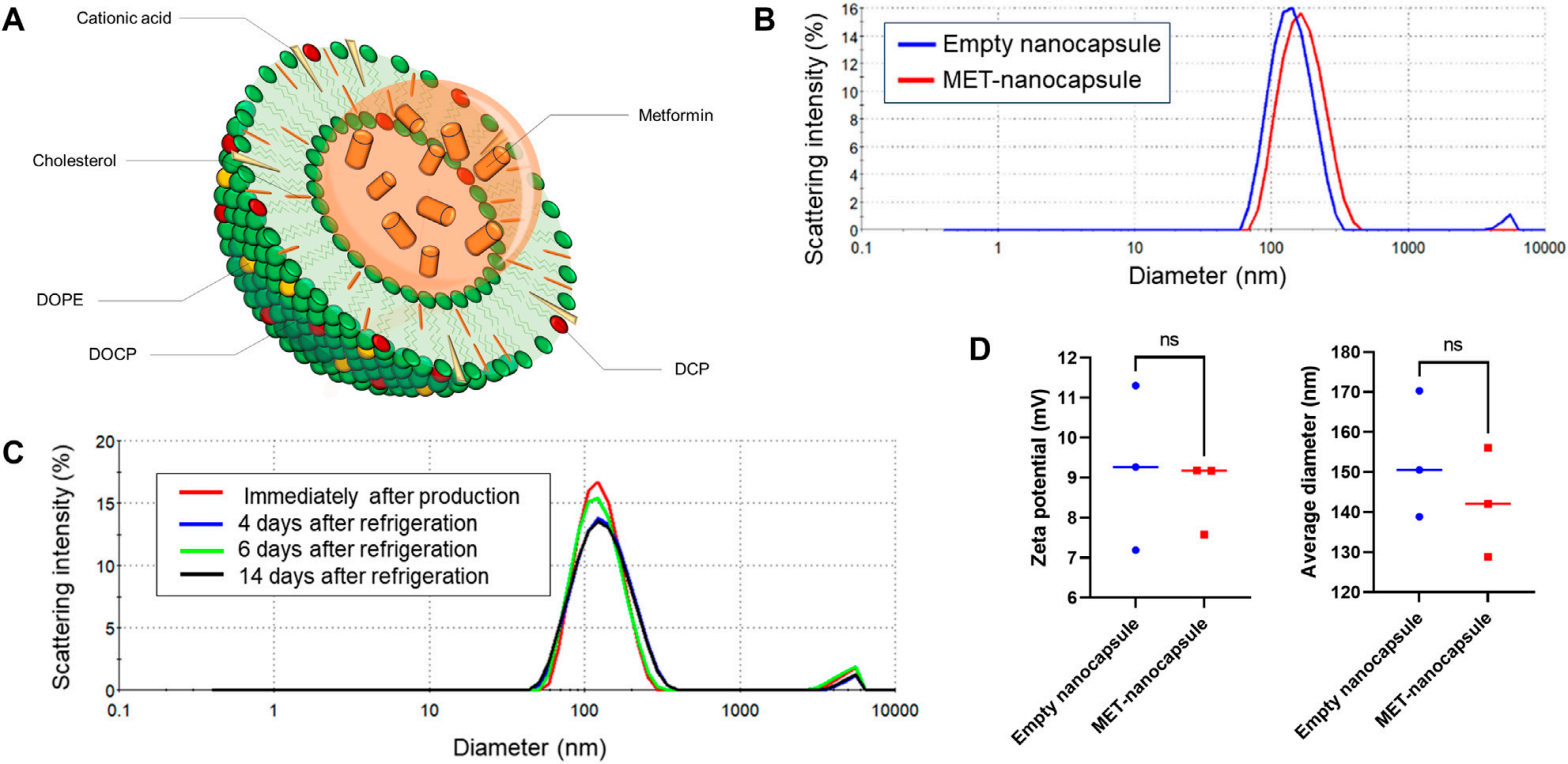
Details of the pulmonary artery-targeted MET-loaded nanocapsules. **(A)** Schematic representation of the nanocapsules. **(B)** Particle size distribution of the empty nanocapsules and MET nanocapsules. **(C)** Storage stability of the nanocapsules. **(D)** Average particle size and zeta potential of the empty nanocapsules and MET nanocapsules (N = 3). Statistical analyses in **(D)** involved unpaired t-test. DOPE, dioleoyl phosphatidylethanolamine; DOCP, dioleoyl phosphatidylcholine; DCP, diocetyl phosphate; MET, metformin; ns, not significant.

The lipid film was hydrated with metformin hydrochloride (250 mg/mL; Fujifilm Wako Pure Chemicals, Osaka, Japan) in 10 mM HEPES buffer (pH 7.4) to achieve a lipid concentration of 50 mg/mL. The hydrated mixture was subjected to three cycles of freezing in liquid nitrogen at −196°C for 10 min, followed by thawing at 40°C for 10 min. Extrusion was performed at 60°C using a Mini-Extruder (Avanti Polar Lipids, Alabaster, AL, United States), which reduced the nanocapsule size from 400 to 200 nm in two steps and further down to 100 nm in two additional steps. Following extrusion, the solution was subjected to tangential flow filtration using a 300-kDa membrane in HEPES buffer (pH 7.2) at 27°C. Finally, the metformin-loaded nanocapsules (MET nanocapsules; Figure 1A) were collected and passed through a 0.2-µm filter (DISMIC-25CS; ADVANTEC, Tokyo, Japan). Several modifications were made to optimize the encapsulation of metformin within the nanocapsules. Initially, owing to metformin’s cationic nature, careful consideration was given to the balance between cationic lipids (dendrimer lipids) and anionic lipids (DCP) in the lipid composition. Furthermore, the metformin concentration in the liposomes was increased through repeated freeze–thaw cycles after introducing the metformin solution to the lipid film. We also increased the concentrations of both lipids and metformin during the preparation phase. Moreover, liposomes were initially prepared without adding sodium chloride to further boost the metformin concentration; sodium chloride was subsequently added during the tangential flow filtration process to remove unencapsulated metformin and achieve isotonic conditions. The storage stability of the nanocapsules was confirmed; after 14 days of refrigeration, there was negligible change in the particle size of the nanocapsules (Figure 1B), and the retention rate of metformin remained at 83% (Figure 1C).

Empty nanocapsules with the same lipid composition were prepared to be used as controls. As shown in Figure 1D, the particle size or zeta potential did not significantly differ between the MET-containing and empty nanocapsules. All nanocapsules were stored at 4°C. Liposome preparation was outsourced to Katayama Chemical Co.

### 2.1 Treatment of human pulmonary arterial smooth muscle cells (hPASMCs) with liposomes and analysis of cell viability

Control hPASMCs were derived from the small vessels (<2 mm diameter) of lung resection specimens of patients with lung carcinoma. None of the patients had pulmonary hypertension or received any chemotherapy or radiation therapy. All hPASMCs were harvested from areas well away from the carcinoma. hPASMCs from patients with PAH (Table 1 for patient details) were obtained from PAH-affected lungs during lung transplantation.

**TABLE 1:** Clinical information of two control individuals and a patient with PAH whose pulmonary smooth muscle cells were used.

Subject ID	Sex	Age (years)	Clinical details
TK4	Male	70	Lung adenocarcinoma
ST5	Female	79	Metastatic lung tumor from liposarcoma
73 MP	Female	30	Heritable PAH (*BMPR2* p.R899X)

PAH, pulmonary arterial hypertension.

The hPASMCs were cultured as previously described (Chida-Nagai et al., 2019) in a humidified 5% CO_2_ incubator at 37°C. Cells from passages five to eight were used. Nanocapsule uptake by hPASMCs was assessed using immunostaining. Empty nanocapsules, labeled with rhodamine-phosphatidylethanolamine (PE; 18:1 Liss Rhod PE Avanti), were administered at a 1:100 dilution to PASMCs from healthy controls, along with Hoechst 33342 (Dojindo, Kumamoto, Japan). After 2 h, the cells were washed three times with TBS-T (50 mM Tris-HCl [pH 7.6], 137 mM NaCl, and 0.1% [w/v] Tween-20) and observed.

To assess the effect of the nanocapsules on cell viability, hPASMCs from both healthy individuals and patients with PAH were cultured and treated with metformin, empty nanocapsules, or MET nanocapsules for 1 h. The medium was subsequently replaced, and this procedure was repeated for five consecutive days. hPASMC viability was assessed using a WST-8 cell proliferation assay kit (Dojindo). This assay relies on the detection of formazan, which is produced by cellular mitochondrial dehydrogenase-mediated cleavage of the tetrazolium salt WST-8.

### 2.2 Cardiac catheterization and evaluation of right ventricular hypertrophy

PAH model rats were generated as previously reported (Abe et al., 2010). Adult male Sprague–Dawley rats (Charles River Laboratories, Yokohama, Japan), weighing 180–220 g, were subcutaneously injected with SU5416 (Tocris; Bioscience, Bristol, United Kingdom) at a dosage of 20 mg/kg. These rats were then exposed to a hypoxic environment (10% O_2_) for 3 weeks in a hypoxic breeding chamber (Shinfactory, Fukuoka, Japan). The rats were then maintained under normal atmospheric conditions for 2 weeks. During this period, metformin (20 mg/kg/dose), empty nanocapsules (total lipid content 120 mg/kg/dose), or MET nanocapsules (20 mg/kg/dose, total lipid content 120 mg/kg/dose) were administered daily via the tail vein. After 2 weeks, the rats received intraperitoneal injections of medetomidine hydrochloride (0.15 mg/kg), midazolam (2 mg/kg), and butorphanol tartrate (2.5 mg/kg), as well as a subcutaneous injection of ketoprofen (2 mg/kg), before being subjected to cardiac catheterization.

The systemic blood pressure of each rat was measured via a catheter inserted from the carotid artery into the aorta. Right ventricular systolic pressure was measured using a polyethylene tube (PE No. 50; Becton Dickinson, Franklin Lakes, NJ, United States) filled with heparinized saline. A 1.4-Fr micromanometer-tipped catheter (Millar Instruments, Houston, TX, United States) was introduced into the polyethylene tube. Data were processed using the LabChart 8 software (ADInstruments, Sydney, Australia). Following hemodynamic analyses, the rats were euthanized with an intraperitoneal injection of pentobarbital (200 mg/kg), and their hearts were excised for right ventricular hypertrophy measurements. The degree of right ventricular hypertrophy was quantified using the Fulton index, which is calculated as the ratio of the weight of the right ventricle to the combined weight of the left ventricle and septum.

### 2.3 Western blotting

After 2 weeks of drug treatment, the rats were euthanized with an intraperitoneal injection of pentobarbital (200 mg/kg). Their lungs were then excised and homogenized in RIPA buffer (Nacalai Tesque, Kyoto, Japan) containing protease and phosphatase inhibitors (Nacalai Tesque). The lung lysates were denatured in sodium dodecyl sulfate (SDS) loading buffer and resolved using 10% SDS-polyacrylamide gel electrophoresis (Bio-Rad, Hercules, CA, United States). The resolved proteins were then transferred onto a polyvinylidene fluoride membrane (The Perfect Membrane, Medium; GenHunter Corporation, Nashville, TN, United States) using the semi-dry blotting method. The membranes were blocked with 5% bovine serum albumin (BSA) and incubated overnight at room temperature with anti-AMP-activated protein kinase (AMPK)-alpha (1:500; 2532S; Cell Signaling Technology, Danvers, MA, United States), anti-phospho-AMPK (1:500; 2531S; Cell Signaling), or anti-ACTB (1:10,000; HPA041271; Sigma-Aldrich) antibodies prepared in 5% BSA. Subsequently, the membranes were incubated with horseradish peroxidase-conjugated goat anti-rabbit IgG (1:10,000; Cell Signaling). Immunoreactive signals were developed using an enhanced chemiluminescence kit (ECL Western blotting Substrate Kit; Abcam, Cambridge, United Kingdom) and visualized using an ImageQuant LAS 4000 system (GE Healthcare, Chicago, IL, United States).

### 2.4 Immunostaining

Lung tissues from rats in each group were trimmed to a thickness of approximately 5 mm and fixed via immersion in 10% neutral-buffered formalin for 2 days. Subsequently, the tissues were paraffin-embedded and sliced to 7-µm-thick sections. These sections were deparaffinized, washed with water, and activated via autoclaving (120°C for 10 min). After washing with TBST, the sections were blocked with 0.3% hydrogen peroxide-methanol solution, rinsed with TBS, and non-specific reactions were blocked with 5% skim milk. The sections were then probed with anti-alpha smooth muscle actin, rabbit monoclonal (1:500; SQab18108; Arigo Biolaboratories, Hsinchu, Taiwan), and rabbit polyclonal proliferating cell nuclear antigen (PCNA; 1:200; ab18197; Abcam) antibodies. Subsequently, the sections were treated with N-Histofine Simple Stain Rat MAX-PO (MULTI) (Nichirei Biosciences Inc., Tokyo, Japan) and developed using 3,3′-diaminobenzidine. The nuclei were stained with Mayer’s hematoxylin, and the sections were cleared with xylene for observation. In addition to immunostaining, Elastica–Masson staining was performed to visualize elastic fibers and collagen.

In each lung section, 10 small pulmonary arteries (diameter: 20–100 μm) were randomly selected, and PCNA-positive cells were counted. Additionally, the medial wall thickness of these small pulmonary arteries was calculated as the medial wall thickness divided by its minor diameter. Small pulmonary arteries were classified as fully muscularized, as previously described (Kikuchi et al., 2018). To determine the percentage of fully muscularized small pulmonary arteries, 10 such arteries were randomly selected from each rat lung and used for evaluation.

### 2.5 *In-vivo* distribution of nanocapsules in rats

Control and PAH model rats were used to assess *in-vivo* nanocapsule distribution. After the induction of PAH via treatment with SU5416 and 10% hypoxia for 21 days, empty rhodamine-PE-labeled nanocapsules (18:1 Liss Rhod PE; Avanti/Sigma-Aldrich) were administered to rats from each group at a total lipid content of 120 mg/kg/dose. The rats were euthanized 24 h after administration, as described above, and the lung, heart, liver, kidney, and spleen were excised. Each organ was fixed via immersion in 10% neutral-buffered formalin for 2 days. After fixation, the organs were washed with PBS and sequentially immersed in 10%, 20%, and 30% sucrose solution under stirring at 4°C overnight. Subsequently, the organs were embedded in OCT compound (Sakura Finetek Japan Co., Tokyo, Japan) and sliced to 7-μm sections. The sections were fixed onto slides and stained with 4′,6-diamidino-2-phenylindole before being observed.

### 2.6 Safety analysis

Blood samples obtained from each group of rats were allowed to coagulate at 4°C. Subsequently, the serum was isolated via centrifugation at 3,000 rpm for 20 min at 4°C. Biochemical parameters measured included the levels of aspartate aminotransferase, alanine aminotransferase, alkaline phosphatase, gamma-glutamyl transferase, blood urea nitrogen, creatinine, total bilirubin, and potassium. These parameters were analyzed at SRL Co. (Tokyo, Japan). Uric acid, lactate, and total ketone bodies were quantified at Oriental Yeast Co. (Tokyo, Japan).

### 2.7 Statistical analysis

Data are expressed as mean ± standard deviation. Statistical analyses were conducted using unpaired *t*-tests or one-way analysis of variance followed by Dunnett’s test, as appropriate. Results with *P*-value <0.05 were considered statistically significant. All statistical analyses were performed using JMP Pro 17 (SAS Institute, Cary, NC, United States).

### 2.8 Human ethics approval and consent to participate

This study was approved by the Institutional Review Board of the Hokkaido University Hospital (approval no.: 018-0306 and 019-0222), in accordance with the tenets of the Declaration of Helsinki. The use of lung tissues from patients with PAH (Ethics ref. 08-H0304-56+5) was approved by the Papworth Hospital Ethics Review Committee. Informed consent was obtained from all participants with lung cancer and PAH according to the regulations of the Declaration of Helsinki.

### 2.9 Animal ethics statement

This study strictly adhered to the ARRIVE guidelines and all animal experiments were conducted in compliance with the Japanese Act on the Welfare and Management of Animals and the NIH Guide for the Care and Use of Laboratory Animals and were approved by the Institutional Animal Care and Use Committee of Hokkaido University (approval no.: 20-0111).

## 3 Results

**FIGURE 2:**
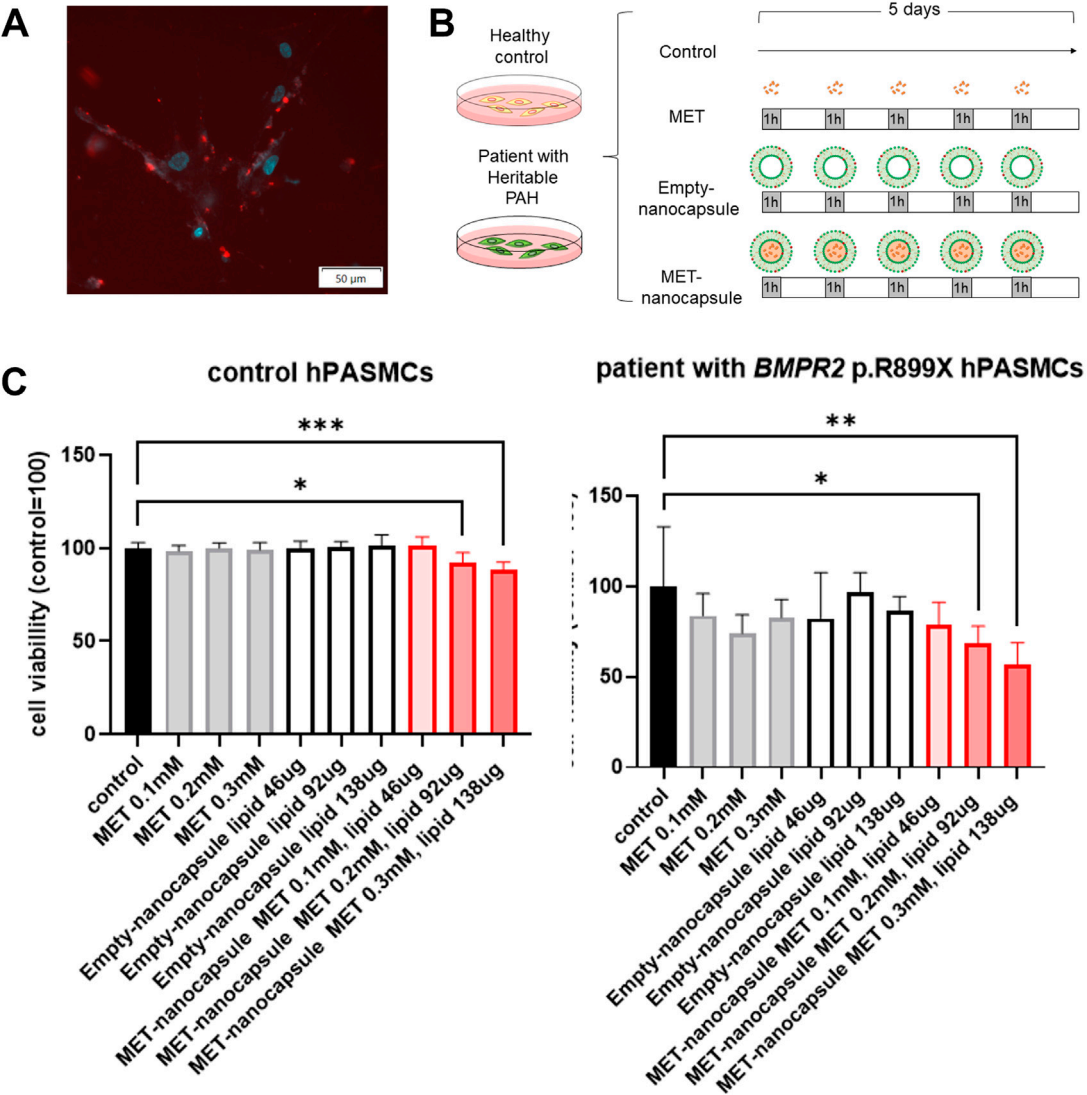
Effects of pulmonary artery-targeted MET nanocapsules on hPASMCs. **(A)** Distribution of fluorescently labeled nanocapsules in hPASMCs. Light blue: Hoechst 33342 (fluorescence wavelength: 510–540 nm); red: rhodamine-PE (fluorescence wavelength: 578 nm). **(B)** Administration protocol of nanocapsules or metformin alone to control hPASMCs and hPASMCs from patients with heritable PAH. **(C)** Cell viability assessment using WST-8. N = 5/group. *P < 0.05, **P < 0.01, ***P < 0.001. Statistical analyses in **(C)** involved the one-way analysis of variance followed by Dunnett’s test. hPASMC, human pulmonary arterial smooth muscle cell; MET, metformin; PAH, pulmonary arterial hypertension.

Rhodamine-PE labeled-empty nanocapsules administered to hPASMCs from healthy controls were effectively internalized into the cytoplasm (Figure 2A).

When metformin, empty nanocapsules, and MET nanocapsules were administered to hPASMCs from healthy controls and patients with heritable PAH at varying concentrations (Figure 2B), we observed that 0.2–0.3 mM MET nanocapsules considerably inhibited the viability of hPASMCs, particularly those from patients harboring *BMPR2* p.R899X (Figure 2C).

**FIGURE 3:**
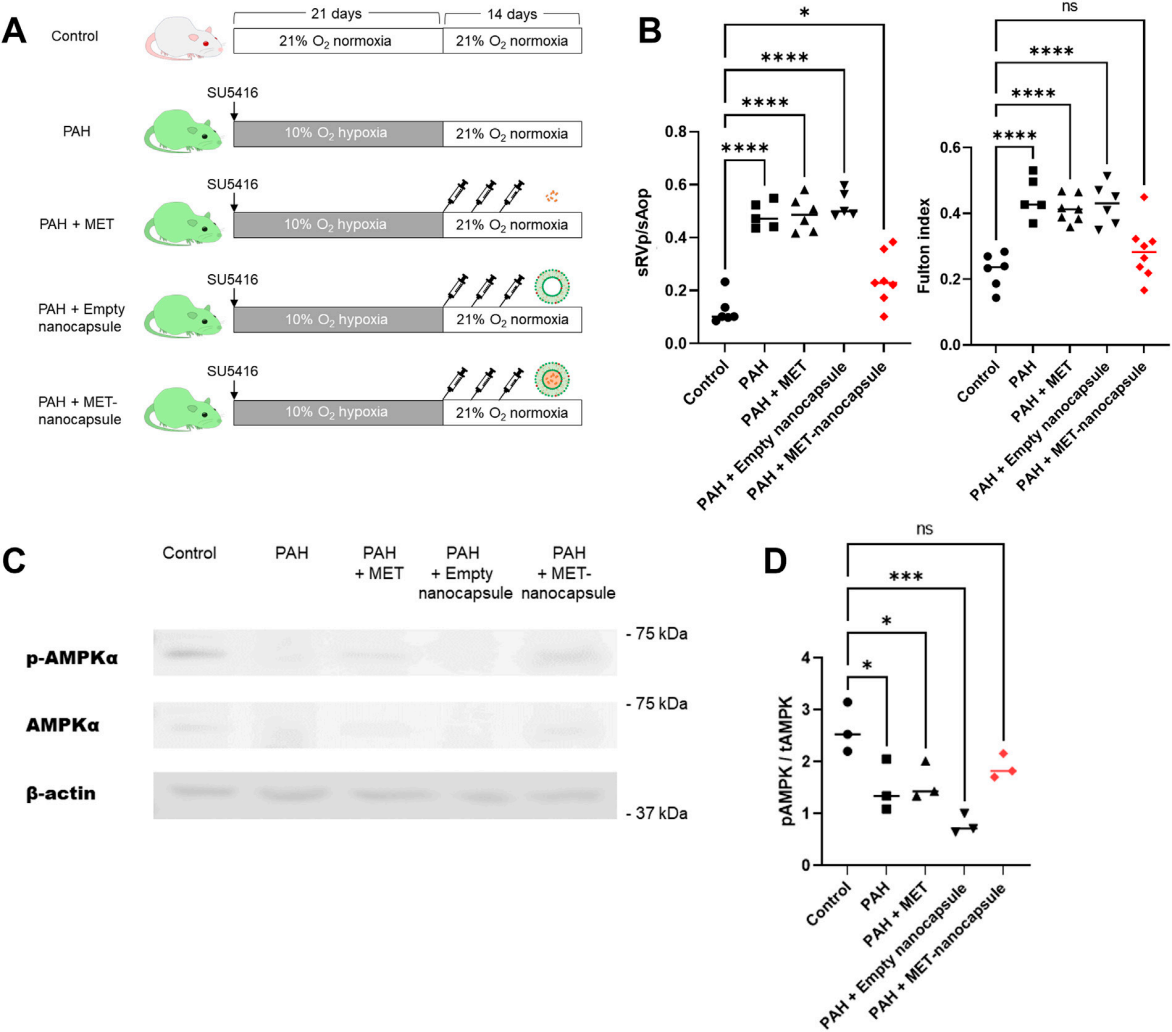
Effects of pulmonary artery-targeted MET nanocapsules in PAH model rats. **(A)** Protocol of administration of nanocapsules or metformin alone to rats in each group. **(B)** Results of cardiac catheterization (systolic right ventricular pressure/systolic aortic pressure. control group N = 6, PAH group N = 5, PAH + MET group N = 6, PAH + Empty nanocapsules group N = 5, PAH + MET nanocapsules N = 7) and the Fulton index (right ventricular weight/left ventricle + ventricular septum weight. control group N = 6, PAH group N = 5, PAH + MET group N = 7, PAH + empty nanocapsules group N = 6, PAH + MET nanocapsules N = 8). **(C)** Western blotting for AMPK and pAMPK expression in the lungs of rats from each group. **(D)** Expression of phosphorylated AMPK is shown relative to that of total AMPK. The values represent mean ± standard deviation from independent experiments (N = 3 per group). *P < 0.05, ***P < 0.001, ****P < 0.0001. Statistical analyses in **(B)** and **(D)** involved the one-way analysis of variance followed by Dunnett’s test. hPASMC, human pulmonary arterial smooth muscle cell; MET, metformin; PAH, pulmonary arterial hypertension; sAop, systolic aortic pressure; sRVp, systolic right ventricular pressure; pAMPK, phosphorylated AMPK; tAMPK, total AMPK; ns, not significant.

**FIGURE 4:**
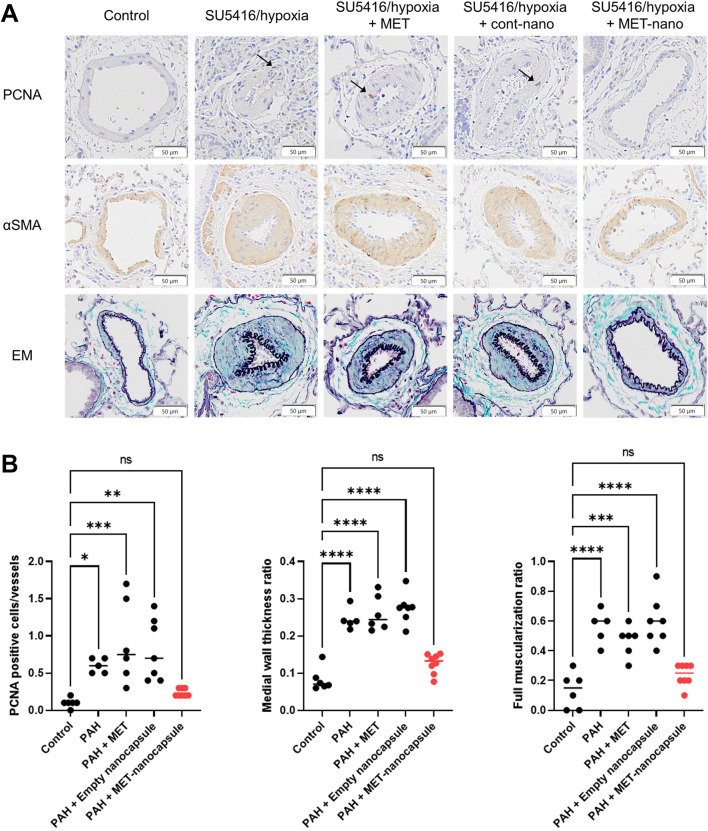
Immunostaining and statistical analysis of the lungs in each rat group. **(A)** Immunostaining of lung sections from rats. **(B)** Number of PCNA-positive cells per 10 small pulmonary arteries, ratio of medial wall thickness to the minor diameter of the small pulmonary arteries, and percentage of fully muscularized small pulmonary arteries per 10 small pulmonary arteries (control group N = 6, PAH group N = 5, PAH + MET group N = 6, PAH + Empty nanocapsules group N = 7, PAH + MET nanocapsules N = 8). *P < 0.05, **P < 0.01. ***P < 0.001, ****P < 0.0001. Statistical analyses in **(B)** involved one-way analysis of variance followed by Dunnett’s test. PCNA, proliferating cell nuclear antigen; αSMA, alpha smooth muscle actin; EM, Elastica–Masson staining; MET, metformin; PAH, pulmonary arterial hypertension.

Control and PAH model rats were treated with metformin, empty nanocapsules, or MET nanocapsules (Figure 3A), and cardiac catheterization was performed. In the PAH model rats, the systolic right ventricular pressure/systolic aortic pressure tended to improve after administration of the MET nanocapsules. In addition, the Fulton index significantly improved in this group (Figure 3B) and the phosphorylated AMP-activated protein kinase (pAMPK)/AMPK ratio was restored (Figure 3C). Western blotting revealed recovery of the ratio of phosphorylated AMPK to total AMPK in the PAH model rats treated with MET nanocapsules (Figure 3D). No significant acute adverse effects were observed when empty nanoparticles or MET nanoparticles were administered to the rats. The immunostaining results of lung sections from rats of each group are presented in Figures 4A, B. In the MET nanocapsule-treated group, significant improvements were observed in the number of PCNA-positive cells, degree of medial hypertrophy, and extent of full small pulmonary artery muscularization.

Serum biochemical testing revealed no significant differences among the treatment groups, and no adverse effects (such as elevated levels of markers of liver or kidney damage, ketosis, or elevated lactate level) were observed upon MET nanocapsule administration (Table 2).

**TABLE 2:** Comparison of serum biochemical parameters among rat treatment groups.

Parameter	Control	PAH	PAH-MET	PAH-empty nanocapsule	PAH-MET nanocapsule	*P* value
AST (IU/L)	302 ± 290	208 ± 107	185 ± 111	267 ± 276	227 ± 96	0.918
ALT (IU/L)	71 ± 45	42 ± 21	40 ± 5.4	42 ± 16	38 ± 12	0.312
ALP (U/L)	81 ± 14	218 ± 70	186 ± 46	166 ± 64	145 ± 49	**0.024**
γGTP (IU/L)	<3	<3	<3	<3	<3	N/A
BUN (mg/dL)	26.2 ± 3.3	32.0 ± 10.0	22.7 ± 4.4	28.7 ± 5.5	21.9 ± 3.0	0.131
Cr (mg/dL)	0.51 ± 0.14	0.52 ± 0.19	0.31 ± 0.06	0.39 ± 0.09	0.29 ± 0.05	**0.037**
T-Bil (mg/dL)	0.11 ± 0.03	0.12 ± 0.06	0.10 ± 0.02	0.11 ± 0.03	0.07 ± 0.02	0.263
potassium (mEq/L)	7.2 ± 2.2	7.5 ± 1.4	5.7 ± 0.8	6.8 ± 0.6	6.8 ± 1.1	0.445
UA (mg/dL)	2.7 ± 2.3	4.1 ± 2.7	2.8 ± 1.6	1.7 ± 0.3	3.7 ± 2.2	0.535
LA (mg/dL)	52.2 ± 34.8	92.9 ± 52.3	44.1 ± 26.9	48.9 ± 13.6	65.3 ± 19.9	0.261
total KB (μmol/L)	557.5 ± 404.5	259.8 ± 78.8	241.3 ± 60.0	236.8 ± 82.8	208.8 ± 55.3	0.109

N = 4 per group. All comparisons were conducted using one-way analysis of variance. Bold values indicate *p* < 0.05.

AST, aspartate aminotransferase; ALT, alanine aminotransferase; ALP, alkaline phosphatase; γGTP, gamma-glutamyl transpeptidase; BUN, blood urea nitrogen; Cr, creatinine; T-Bil, total bilirubin; UA, uric acid; LA, lactic acid; KB, ketone body.

**FIGURE 5:**
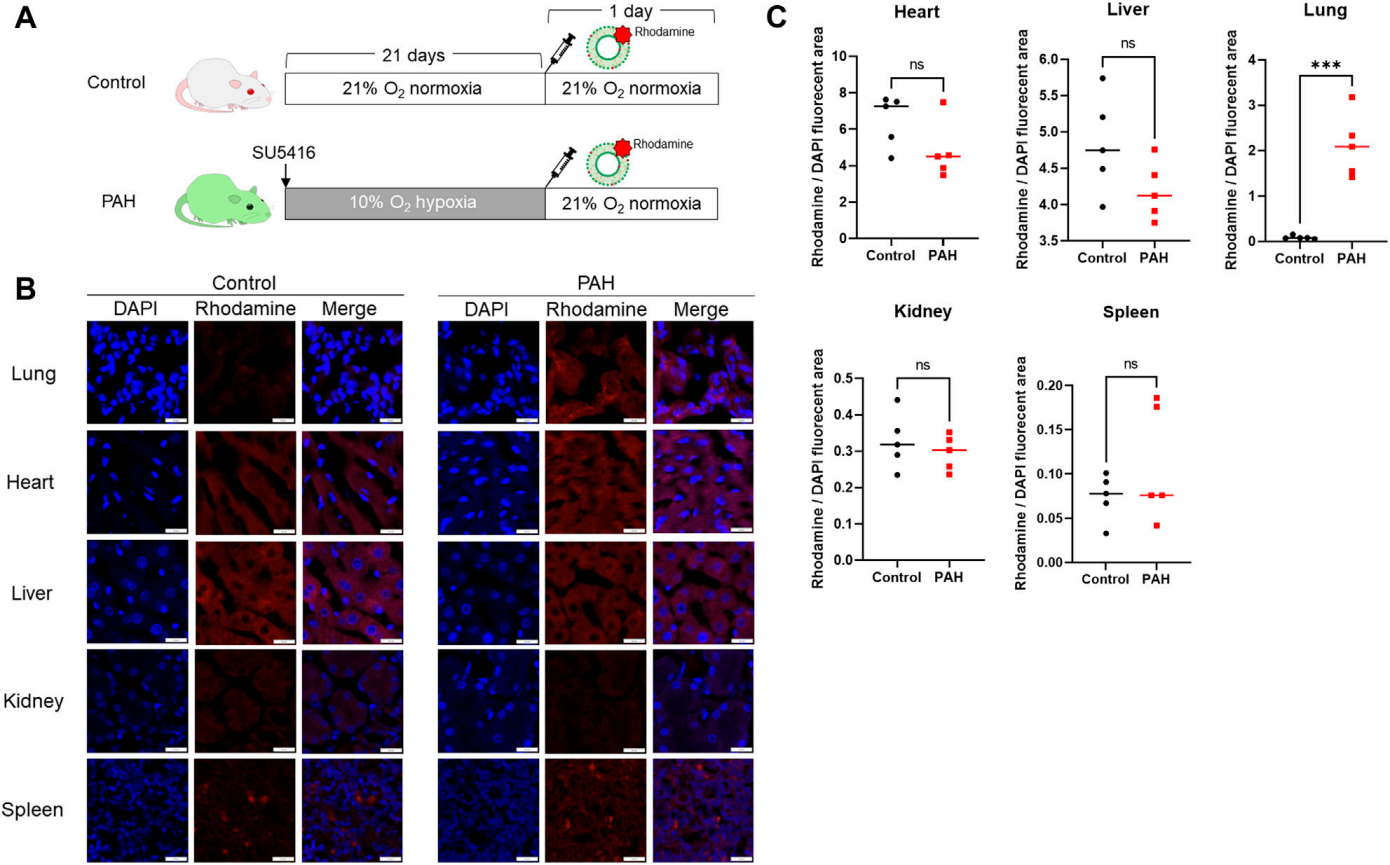
*In-vivo* distribution of pulmonary artery-targeted nanocapsules. **(A)** Protocol for the administration of rhodamine-PE-labeled nanocapsules to control (N = 5) and PAH model rats (N = 5). **(B)** Fluorescent staining demonstrating the distribution of fluorescently labeled nanocapsules in five organs. **(C)** Ratio of the rhodamine-positive to 4′,6-diamidino-2-phenylindole-stained areas in each organ. ***P < 0.001. Statistical analyses in **(C)** involved unpaired *t*-tests. PE, phosphatidylethanolamine.

Finally, we investigated the *in-vivo* distribution of the lung-targeted nanocapsules (Figure 5A). Rhodamine-labeled-empty nanocapsules were administered to control and PAH rats, and their distribution in the lungs, heart, liver, kidneys, and spleen was examined. The nanocapsules significantly accumulated in the lungs of PAH rats compared with that in the control group (Figures 5B, C).

## 4 Discussion

In this study, low-dose metformin-loaded pulmonary artery-targeted nanocapsule administration suppressed PASMC proliferation in a rat model of SU5416+hypoxia-induced PAH, leading to improvements in hemodynamics and right ventricular hypertrophy, thereby slowing the progression of PAH.

Some studies have suggested the potential utility of metformin, an antidiabetic agent, in PAH treatment (Brittain, et al., 2020; Dean, et al., 2016). Metformin reportedly improves right ventricular function and combats oxidative stress in patients with PAH as well as in rodent PAH models (Brittain, et al., 2020).

In this study, we demonstrated that by encapsulating a minimal amount of metformin in nanocapsules, its effectiveness in alleviating PAH could be improved without major adverse effects.

In previous studies, metformin was administered to PAH model rats at a daily dose of 100–150 mg/kg/day for 2–3 weeks (Abdelazeem et al., 2023; Dean, et al., 2016). In comparison, our nanoparticle method enabled almost 80% reduction in the amount of metformin administered.

Liposome nanoparticles have been employed as relatively novel drug delivery systems for targeted organ delivery (Meng et al., 2022). Based on previous reports, we hypothesized that the presence of cationic peptides is necessary to achieve selective accumulation of nanocapsules within the pulmonary arteries (Conary et al., 1994; Kusumoto et al., 2013; Maus et al., 1999). To mitigate the cytotoxicity that accompanies strong cationic compounds, we incorporated negatively charged DCP. We further determined that intravenous administration would be the most appropriate approach for achieving effective delivery to the pulmonary arteries.

The nanocapsules accumulated at high levels in the lungs of PAH model rats compared with those in control rats. Nanoparticles typically exhibit enhanced permeability and retention in inflammatory tissues, enabling their extravasation from blood vessels and prolonged action in these tissues (Barua and Mitragotri, 2014). In the present study, the inflammation present in the small pulmonary arteries in PAH likely facilitated nanocapsule accumulation.

Studies have suggested the effectiveness of metformin in animal models of pulmonary fibrosis (Teague et al., 2022; Wu et al., 2022), which is particularly relevant because pulmonary fibrosis-associated pulmonary hypertension is frequently observed in adults (Ghigna et al., 2017). In addition, Moral-Sanz et al. (2022) used PASMCs to demonstrate the role of AMPK deficiency in the development of persistent pulmonary hypertension in newborns. The MET nanocapsules developed in our study may be effective for PAH and other types of pulmonary hypertension. Additionally, we observed no signs of hypoglycemia, liver impairment, or kidney dysfunction after the administration of the MET nanocapsules, suggesting their safety in newborns, infants, and older individuals. The lipid bilayer developed in this study can potentially enhance the therapeutic effects of established pulmonary arterial vasodilators and novel candidates for PAH treatment via encapsulation. Therefore, our nanoparticles may have extended application potential in PAH treatment.

This study had some limitations. First, only one type of PAH rat model was used, and the sample size was limited. Future studies should validate the efficacy of our nanocapsules in additional PAH animal models with larger sample sizes. Second, the treatment and observation periods in this study were short. Evaluating whether MET nanocapsules improve long-term outcomes in PAH model rats or assessing their long-term safety should be conducted in future studies. Moreover, for practical applications, methods for large-scale production and quality assurance measures need to be developed. Additionally, further pharmacokinetic studies on absorption, metabolism, and excretion are needed. In this study, we demonstrated that the PAH + MET nanoparticle group achieved similar hemodynamics and Fulton index values to healthy rats of the same strain. However, this does not represent a direct comparison between PAH and PAH + MET. In Table 2, the reason for the significant difference observed in alkaline phosphatase and creatinine remains unclear. However, we believe this does not affect the safety of the nanocapsules during the observation period.

The intravenous administration of MET nanocapsules is a safe and innovative therapeutic approach for PAH model rats. This method shows potential for enhancing treatment outcomes while minimizing adverse effects and may also be applicable to other forms of pulmonary hypertension.

## Data Availability

The raw data supporting the conclusions of this article will be made available by the authors, without undue reservation.

## Abbreviations

AMPK, AMP-activated protein kinase; BSA, bovine serum albumin; DCP, diocetyl phosphate; DOPE, dioleoyl phosphatidylethanolamine; hPASMC, human pulmonary arterial smooth muscle cell; Met, metformin; PE, phosphatidylethanolamine; pAMPK, phosphorylated AMP-activated protein kinase; PCNA, proliferating cell nuclear antigen; PAH, pulmonary arterial hypertension; SDS, sodium dodecyl sulfate.
